# Supplementary material for: CHDbase: A Comprehensive Knowledgebase for Congenital Heart Disease-related Genes and Clinical Manifestations
Source: Genomics Proteomics Bioinformatics. 2022 Aug 10;21(1):216–27. doi: 10.1016/j.gpb.2022.08.001 (PMC10372913; doi:10.1016/j.gpb.2022.08.001)
Supplement: Supplementary File S1 — Data collection and analyses [file mmc1.docx]

**File S1 Data collection and analyses**

**Literature search**

We searched the PubMed database to obtain publications related to congenital heart disease (CHD) using the query term “(congenital heart disease*[All Fields] OR heart defect*[All Fields] OR transposition of the great arteri*[All Fields] OR pulmonary atresia[All Fields] OR pulmonary artery atresia[All Fields] OR Anomalous pulmonary venous*[All Fields] OR Ebstein anomaly[All Fields] OR Epstein anomaly[All Fields]) AND (gene[Title/Abstract] AND (proteomics[Title/Abstract] OR expression[Title/Abstract] OR CNV[Title/Abstract] OR copy number variation[Title/Abstract] OR microarray*[Title/Abstract] OR microdel*[Title/Abstract] OR microdup*[Title/Abstract] OR rearrange*[Title/Abstract] OR linkage[Title/Abstract] OR associa*[Title/Abstract] OR scan[Title/Abstract] OR sequenc*[Title/Abstract])) AND (“1000/01/01”[Date - Publication]: “2020/01/10”[Date - Publication])”. The abstracts of 2762 studies retrieved were then carefully reviewed to remove irrelevant papers, and the remaining 1114 studies were subjected to systemic curation.

**Network-based CHD gene prioritization**

We developed an unweighted network for 1124 CHD-related genes based on protein-protein interactions from STRING database (https://string-db.org) [[1](#_ENREF_1)]. Only experimentally-verified protein-protein interactions from STRING were used. Specifically, in the network, the two genes were connected with an edge only when the STRING experimental score > 0. Next, we used three centralities to measure the significance of each gene in the network, including the degree, the betweenness centrality, and the eigenvector centrality [[2](#_ENREF_2)]. These parameters were calculated in R package “igraph”.

We then used *k*-core decomposition to define the core nodes of the network, and extracted the core sub-network from a large network [[3](#_ENREF_3)]. Specifically, *k*-core decomposition is started by removing all nodes with degree *k* = 1 from a network. This process may cause new nodes with degree *k* ≤ 1, which are also removed until the degree *k* of all the remaining nodes > 1. The removed nodes and their links during the process of *k* = 1 form the 1-shell. Next, this kind of pruning process is continued by *k* = 2 to extract 2-shell and repeated until all higher-layer shells are extracted and all nodes in the network are removed. In practice, we used the function “coreness()” to identify the *k* score of each node. Then the nodes with the highest *k* score (*k* = 70) were defined as core nodes of the network, and the sub-network of these core nodes was defined as the core sub-network. The network was then visualized with the Cytoscape tool (Version 3.8.2) [[4](#_ENREF_4)]. To evaluate the confidence of the core genes in the *k*-core, we compared the difference between the core genes and all of the 1124 CHD-related genes in the number of supporting evidence items using a two-sided Mann–Whitney U test.

**Expression profile analysis**

We collected RNA-Seq data for different developmental time points in the human brain and heart from the European Bioinformatics Institute submitted by Cardoso-Moreira and his colleagues (E-MTAB-6814, https://www.ebi.ac.uk) [[5](#_ENREF_5)]. Tissue- and time-specificity indexes were also obtained from the study of Cardoso-Moreira and his colleagues [[5](#_ENREF_5)]. The expression profiles, tissue- and time-specificity indexes of different gene categories were identified and compared with Mann–Whitney U test.

**CHD classification**

Copy number variations and linkage regions associated with the disease typically cover numerous genes, making it difficult to identify true causal genes. We thus excluded copy number variation and linkage data when performing CHD classification. We further removed single-nucleotide variants/insertion-deletion variants (SNVs/Indels) that were classified as “benign”, “likely benign”, and “uncertain” by the original publications, as the authors did not draw a concrete conclusion about the association.

We then calculated the pairwise Jaccard coefficient and statistical significance for the 27 most frequently reported CHD types associated with at least ten genes in CHDbase, using the Jaccard.test function of the Jaccard R package with the measure concentration algorithm. The Jaccard coefficient is defined as the number of shared genes divided by the total number of unique genes of two CHD types. We further obtained adjusted *P* values by correcting for multiple testing using the Benjamini–Hochberg (BH) false discovery rate (FDR). Based on the matrix of Jaccard distance, which equals 1 minus the Jaccard coefficient, we performed hierarchical clustering analysis to classify the 27 CHD types into homogeneous groups using the pheatmap R package with the clustering method of ward.D. To be conservative, we reclassified the variants in CHDbase using InterVar (Version: 2.2.2) [[6](#_ENREF_6)]. A total of 21 variants considered as “disease causing” or “likely disease causing” by the original publications were reclassified as “benign” or “likely benign” due to high frequency in population data. We then repeated the above analyses after removing these 21 variants and compared the results with the previous findings.

**References**

[1] Szklarczyk D, Gable AL, Lyon D, Junge A, Wyder S, Huerta-Cepas J, et al. STRING v11: protein-protein association networks with increased coverage, supporting functional discovery in genome-wide experimental datasets. Nucleic Acids Res 2019;47:D607–13.

[2] Newman MEJ. Networks: an introduction. Oxford: Oxford University Press; 2010.

[3] Dorogovtsev SN, Goltsev AV, Mendes JF. *k*-core organization of complex networks. Phys Rev Lett 2006;96:040601.

[4] Su G, Morris JH, Demchak B, Bader GD. Biological network exploration with Cytoscape 3. Curr Protoc Bioinformatics 2014;47:8.13.1–24.

[5] Cardoso-Moreira M, Halbert J, Valloton D, Velten B, Chen C, Shao Y, et al. Gene expression across mammalian organ development. Nature 2019;571:505–9.

[6] Li Q, Wang K. InterVar: clinical interpretation of genetic variants by the 2015 ACMG-AMP guidelines. Am J Hum Genet 2017;100:267–80.
